# Senile dementia and psychiatric stigma among community health service providers and relatives of diagnosed and suspected dementia patients: a cross-sectional study

**DOI:** 10.7717/peerj.14613

**Published:** 2023-01-11

**Authors:** Qiwen Zhang, Jing Deng, Huanyue Luo, Li Wang

**Affiliations:** 1 Department of Geriatric Psychiatry, The Second People’s Hospital of Guizhou Province, Guiyang, Guizhou, China; 2 Department of Psychiatry, The Second People’s Hospital of Guizhou Province, Guiyang, Guizhou, China; 3 Department of VIP Clinic, The Second People’s Hospital of Guizhou Province, Guiyang, Guizhou, China

**Keywords:** Public health, Dementia, Suspected dementia, Health service use, Knowledge, Psychiatric stigma, Health service providers, Relatives, Community, Delayed diagnosis

## Abstract

**Background:**

The number of people suffering from dementia is increasing rapidly in China. Early identification, referral, and intervention for dementia patients within communities are important to public health. However, these measures could be impacted by misconceptions about dementia and associated psychiatric stigma from community health professionals and relatives of dementia patients.

**Methods:**

A cross-sectional survey was conducted on 249 participants, which included community doctors, community nurses, and relatives of diagnosed and suspected dementia patients in Guiyang, China. Participants were recruited through convenient sampling. The Chinese version of Dementia Knowledge Assessment Scale (DKAS) and the Perceived Psychiatric Stigma Scale (PPSS) were used to evaluate the participants’ knowledge of dementia and dementia-related psychiatric stigma.

**Results:**

A total of 249 participants completed the questionnaire. The participants had moderate overall knowledge of dementia and the associated psychiatric stigma. Participants who were ≥45 years old, had a low level of education, had a low monthly income, or gained knowledge of dementia through non-media channels had lower awareness of dementia and stronger psychiatric stigma. In the “Communication & behavior” subscale of DKAS, all participants had a low level of awareness. Relatives of diagnosed and suspected dementia patients had higher total PPSS and “Marital preclusion” subscale scores than community doctors and nurses but lower psychiatric stigma based on the PPSS “Self-deprecation” subscale score.

**Conclusions:**

Despite their profession, community doctors and nurses did not show an absolute advantage over relatives of diagnosed and suspected dementia patients in the dementia knowledge, and they even showed higher psychiatric stigma in some subscales. The self-deprecation subscale is related to the identification with negative labels such as “people with a mental illness are the weak”. This study shows that reducing stigma on the “Self-deprecation” subscale should be a core component of training and educational programs targeted at improving dementia knowledge among community health service providers.

## Introduction

The aging of the global population has led to a rapid increase in the number of dementia patients (Davis et al., 2021). The number of dementia-afflicted people has increased by 117% globally in the last three decades (Nichols et al., 2022). About 50 million people worldwide suffer from dementia, more than two-thirds of whom live in low- or middle-income countries (Livingston et al., 2020; Mattap et al., 2022). A study published in *The Lancet Public Health* in 2020 reported there were over 15 million dementia patients in China (Jia et al., 2020). Jia et al. (2018) estimated that by 2020, medical expenses of dementia patients in China would be as high as 250 billion US dollars, accounting for nearly one-fifth of global dementia-related costs. It has been estimated that by 2050, the number of dementia patients in China will reach 45 million, and the annual treatment cost will reach 1.89 trillion US dollars (Jia et al., 2018; Nichols et al., 2022). Dementia has become the third most serious health problem in the world after cancer and cardiovascular and cerebrovascular diseases, the fifth leading cause of death in China and the sixth in the United States (Epps et al., 2020; Ren et al., 2021). Dementia poses significant social and economic challenges to social policy development and health service delivery (Fang et al., 2020).

The diagnosis and treatment of dementia are seriously insufficient throughout the world, with diagnosis rates being only about 50% in high-income countries and 5%–10% in low- and middle-income countries (Lian et al., 2017). In China, the diagnosis rate of dementia is only about 25%, the rate of missed diagnosis is as high as 76.8%, and more than 90% of dementia cases remain undetected in communities (Tian et al., 2019). Although dementia cannot be cured, substantial evidence shows that early diagnosis is beneficial (Livingston et al., 2017), and medical staff in primary medical institutions play an important “gatekeeper” role in the prevention and treatment of dementia in communities (Tierney et al., 2019). The significant underutilization of health services by dementia patients adds to the importance of our research topic (Prince, Bryce & Ferri, 2011; Bradford et al., 2009; Lee et al., 2019). This study investigates the knowledge of and psychiatric stigma associated with senile dementia among community health professionals and relatives of diagnosed and suspected dementia patients in Guiyang (the capital of Guizhou province of China) to reflect corresponding awareness and psychiatric stigma amongst community health service providers, health service users, and potential health service users. The gap between China and other countries could also be reflected.

National awareness campaigns must tackle stigma and lack of education on dementia, as they continue to be barriers to diagnosis and seeking post-diagnosis support (Gauthier et al., 2022). Since behavioral and psychological symptoms of dementia (BPSD) overlap with those of certain psychiatric disorders (*e.g.*, schizophrenia), people unfamiliar with the characteristics of dementia may perceive these patients as “psychopaths” and make false attributions (Su & Chang, 2020). In recent decades, the psychiatric stigma of severe mental disorders, such as schizophrenia and bipolar affective disorder, has received much attention worldwide, but there has been limited research on the psychiatric stigma of dementia (Lee, Hong & Casado, 2021). There is no consensus on how to better assess the stigma associated with dementia, and most studies have been conducted in developed countries in Europe and America, with few reports from low- and middle-income countries. Due to social and cultural differences, the results of these studies may not be universally applicable in China and other low- and middle-income countries (Wang et al., 2018).

In recent years, a number of studies have been conducted on the knowledge and stigma associated with dementia in different populations, including local residents, families of dementia patients, and medical staff (Velilla et al., 2022; Kim et al., 2019; Owokuhaisa et al., 2020). Most studies are limited to a specific group, and only a few of them investigate multiple groups simultaneously in a period of time. Most of the results are difficult to be compared horizontally because they were based on various research tools (Ayisi-Boateng et al., 2022; Strøm, Engedal & Andreassen, 2019; Crawley et al., 2022; Oliveira et al., 2022).

Community-based services improve the quality of life for dementia patients and their caregivers (Morgan et al., 2015). China has a low prevalence of knowledge of dementia, which is viewed as a normal part of the aging process, and the behavioral and cognitive symptoms of dementia are attributed to mental illnesses (Liu et al., 2008). A recent study showed that more than a quarter of the rural elderly in China is at a high risk of dementia (Cong et al., 2022). However, dementia has not yet received enough attention at the national level in China, and a national strategic action plan for dementia has not been considered in the “Healthy China 2030” Plan and China’s Medium- and Long-term Plan for the Prevention and Treatment of Chronic Diseases (2017–2025) (Wang et al., 2022). There is a general lack of dementia knowledge and training in community hospitals in China, and only a few medical staff actually participate in diagnosis, treatment and nursing work. Therefore, community medical staff may not have more knowledge about dementia than the relatives of those suffering from dementia, and relatives play an important role in their decision to seek diagnostic evaluation when dementia is suspected (Helvik et al., 2018). Further research is needed to reduce delayed visits, referrals, diagnosis, treatment and recovery caused by the lack of dementia knowledge and psychiatric stigma in the community. This research can also provide evidence for the international community to support the timely diagnosis, treatment, and rehabilitation of dementia patients in community settings. To the best of our knowledge, this is the first cross-sectional survey of dementia knowledge and psychiatric stigma in China, based on the community, among the providers, users, and potential users of community health services.

## Materials & Methods

### Study design

A cross-sectional survey was conducted among community health professionals and relatives of diagnosed and suspected dementia patients to collect self-reported knowledge of dementia and perceived psychiatric stigma.

### Sample

The survey was conducted in Guiyang, Guizhou Province, China. Guiyang is the capital and the third most populous city of Guizhou Province, with a population density of 745 people per square kilometer. It consists of six districts, three counties, and one county-level city. The numbers of medical and health technicians and medical practitioners or assistants per thousand population were 10.97 and 3.98 (7.38 and 2.48 in the province) (Statistics Bureau of Guizhou Province, 2020). The seventh national population census shows that the 60-and-over population in Guiyang accounts for 13.3% of the city’s total population, indicating that Guiyang is becoming an advanced aging society (Guiyang Municipal Bureau of Statistics, 2020).

Our research team stayed in Guiyang from June 2019 to December 2020 to conduct the questionnaire survey and collect data. Before the investigation, based on the reference (Annear et al., 2017), the Dementia Knowledge Assessment Scale (DKAS) scoring rate *π* was set at 0.60 for different populations in the community, and Z_*α*/2_ = 1.96 under the conditions of allowable error *δ* 0.1, lost follow-up rate 0.15, and test level *α* = 0.05. According to the formula *n* = (Z_*α*/2_/ *δ*) ^2^ *π* (1- *π*)/(1−0.15), the sample size was set to be 300. Two districts (Nanming District and Guanshanhu District) and one county (Xifeng County) were randomly selected from six districts and three counties of Guiyang. In each district/county, 30 community doctors, 30 community nurses, 20 family caregivers of dementia patients and 20 relatives of suspected dementia patients were investigated. Then, a questionnaire survey was conducted among the medical staff in community health service centers or stations, township health centers, village clinics and private clinics, and relatives of diagnosed and suspected dementia patients in the community of the selected area by means of convenient sampling. Inclusion criteria: (1) Doctors and nurses in community health service centers or stations, township health centers, village health clinics, and private clinics (excluding those having family members with diagnosed dementia or suspected dementia); (2) primary family caregivers (for at least three months) of persons aged 60 or above in the community who have been diagnosed with dementia in a hospital at or above the county level; (3) primary family caregivers (living together for at least six months) of persons aged 60 or above in the community who are suspected of dementia but have never sought medical treatment; (4) those who gave informed consent and volunteered to participate in this study; (5) clear consciousness and speech; (6) respondents living in the community for at least six months at the time of investigation. Exclusion criteria: (1) Respondents with a history of mental illness; (2) respondents with serious cognitive impairment such as dementia and those who refused to participate.

### Demographic data

We designed the demographic questionnaire that covers gender, age, education level, marital status, per capita monthly household income, work status, and place of residence.

### Evaluation of the cognitive function of suspected dementia patients

A Chinese version of the AD8 questionnaire was used by family members (relatives) to assess the cognitive dysfunction of suspected dementia patients who were over 60 years but had never sought medical treatment. A result score ≥2 is considered to indicate cognitive impairment. People with a past history of mental illness were excluded. The total score of the Hamilton Depression Scale (24-item version) assessed by geriatric psychiatrists was less than 8, and the value of the Clinical Dementia Rating (CDR) was 0.5. AD8 is a self-rating cognitive impairment screening tool. Galvin et al. (2005) from the University of Washington summarized 55 questionnaire items based on literature in 2002. After 290 trials, the official edition of AD8 was published by them in 2005 with eight items to assess changes in patients induced by cognitive problems. Li et al. (2012) conducted a reliability and validity study on the Chinese version of the AD8 scale. The results showed that the AUC of people with normal cognition and those with AD was 0.93. The sensitivity was 93.9%, and the specificity was 76.0%, with a cognitive impairment cutoff score of 2, which implied good reliability and validity.

### Dementia Knowledge Assessment Scale

The amount of knowledge that community doctors, community nurses, and relatives of diagnosed and suspected dementia patients had on dementia was assessed using the Dementia Knowledge Assessment Scale (DKAS). The scale was developed by Annear et al. (2015), researchers from the dementia research and education center in Australia in 2015. It is used to measure dementia knowledge and reflect broader information related to dementia. DKAS has been shown to be a reliable and valid measure of dementia knowledge across a range of participant populations (Tierney et al., 2019). Zhang (2018) translated this scale into Chinese. As shown in Table 1, the scale comprises 25 items across four subscales: “Causes & characteristics” (seven items), “Communication & behavior” (six items), “Care considerations” (six items), and “Risks & health promotion” (six items). Each item is scored from 0 to 2 according to the five possible answers to the item: true, possibly true, false, possibly false, and don’t know. For true knowledge items, “true” answers are scored with 2 points, “probably true” with 1 point, and “false”, “probably false”, and “don’t know” with 0. For false knowledge items, “false” answers are scored with 2 points, “probably false” with 1 point, and “true”, “probably true”, and “don’t know” with 0. The sum of these scores ranges from 0 to 50, and higher scores indicate better knowledge. This scale is suitable for the assessment of dementia knowledge among health service workers and family caregivers who have not been trained in dementia (Sung et al., 2021; Carnes et al., 2021; Zhang, 2018). In this study, the Cronbach’s alpha internal consistency reliability coefficient of DKAS was 0.796, and those of “Causes & characteristics”, “Communication & behavior”, “Care considerations”, and “Risks & health promotion” were 0.652, 0.705, 0.690, and 0.674, respectively.

**Table 1:** DKAS and confirmed domains.

Item	Statement content	Confirmed domains
1	Most forms of dementia do not generally shorten a person’s life [*False* ]	Causes & characteristics
2	Blood vessel disease (vascular dementia) is the most common form of dementia [*False* ]	Causes & characteristics
3	People can recover from the most common forms of dementia [*False* ]	Causes & characteristics
4	Dementia is a normal part of the ageing process [*False* ]	Causes & characteristics
5	Dementia does not result from physical changes in the brain [*False* ]	Causes & characteristics
6	Planning for end of life care is generally not necessary following a diagnosis of dementia [*False* ]	Causes & characteristics
7	Alzheimer’s disease is the most common form of dementia [*True* ]	Causes & characteristics
8	It is impossible to communicate with a person who has advanced dementia [*False* ]	Communication & behavior
9	A person experiencing advanced dementia will not generally respond to changes in their physical environment [*False* ]	Communication & behavior
10	It is important to correct a person with dementia when they are confused [*False* ]	Communication & behavior
11	People experiencing advanced dementia often communicate through body language [*True* ]	Communication & behavior
12	Uncharacteristic behaviors in a person experiencing dementia are generally a response to unmet needs [*True* ]	Communication & behavior
13	Medications are the most effective way of treating behavioral symptoms of dementia [*False* ]	Communication & behavior
14	Movement is generally affected in the later stages of dementia [*True* ]	Care considerations
15	Difficulty eating and drinking generally occurs in the later stages of dementia [*True* ]	Care considerations
16	People with advanced dementia may have difficulty speaking [*True* ]	Care considerations
17	People experiencing dementia often have difficulty learning new skills [*True* ]	Care considerations
18	Daily care for a person with advanced dementia is effective when it focuses on providing comfort [*True* ]	Care considerations
19	People experiencing dementia do not generally have problems making decisions [*False* ]	Care considerations
20	Having high blood pressure increases a person’s risk of developing dementia [*True* ]	Risks & health promotion
21	Maintaining a healthy lifestyle does not reduce the risk of developing the most common forms of dementia [*False* ]	Risks & health promotion
22	Symptoms of depression can be mistaken for symptoms of dementia [*True* ]	Risks & health promotion
23	The sudden onset of cognitive problems is characteristic of common forms of dementia [*False* ]	Risks & health promotion
24	Exercise is generally beneficial for people experiencing dementia [*True* ]	Risks & health promotion
25	Early diagnosis of dementia does not generally improve quality of life for people experiencing the condition [*False* ]	Risks & health promotion

### Perceived Psychiatric Stigma Scale

The Perceived Psychiatric Stigma Scale (PPSS), a scale developed by Han & Chen (2008), was used to accurately assess dementia-related psychiatric stigma among community doctors, community nurses, and relatives of diagnosed and suspected dementia patients. As shown in Table 2, there are 25 items across three subscales: “Social ostracism” (eleven items), “Marital preclusion” (eight items), and “Self-deprecation” (six items). A four-point Likert scale was used to score the scale items, with “1” representing “completely disagree”, “2” representing “disagree”, “3” representing “agree”, and “4” representing “completely agree”. Scores of each item are positive, and the higher the score, the stronger the perceived psychiatric stigma. “Social ostracism” subscale scores range from 11 to 44, “Marital preclusion” scores range from 8 to 32, and “Self-deprecation” scores range from 6 to 24 to give a total score that ranges from 25 to 100. The scale is suitable for evaluating the perceived psychiatric stigma of mentally ill and healthy people. Participants should imagine how they would be treated by others if they were a relative or a primary caregiver for a dementia patient with BPSD. In this study, the Cronbach’s alpha internal consistency reliability coefficient of PPSS was 0.724, and those of “Social ostracism”, “Marital preclusion”, and “Self-deprecation” were 0.742, 0.791, and 0.722, respectively.

**Table 2:** PPSS and confirmed domains.

Item	Statement content	Confirmed domains
1	If I suffer from a mental illness, I will be judged morally deficient.	Social ostracism
2	I don’t think anyone wants to marry someone who is mentally ill.	Marital preclusion
3	I’d be embarrassed to see a psychiatrist.	Social ostracism
4	People with a mental illness are weak.	Self-deprecation
5	If people know I was seeing a psychiatrist, it would ruin my relationships.	Social ostracism
6	If I have a mental illness, I feel inferior to others.	Self-deprecation
7	Seeing a psychiatrist is a sign of mental ill health.	Self-deprecation
8	It’s not good to tell people about my own mental problems.	Social ostracism
9	If I have a mental illness, people will think that there is also something wrong with my ancestors.	Social ostracism
10	Psychopaths are ostracized by the parents of the people they marry.	Marital preclusion
11	If I have a mental illness, my family would feel humiliated.	Social ostracism
12	If I’m mentally ill, it means I’m not a “normal person”.	Self-deprecation
13	If I tell people I was seeing a psychiatrist, they wouldn’t accept it.	Social ostracism
14	Seeing a psychiatrist would leave a bad record on my life.	Social ostracism
15	If I have a mental illness and it is known by others, my reputation would be damaged.	Social ostracism
16	Seeing a psychiatrist means I’m not a strong person.	Self-deprecation
17	People who have suffered from mental illnesses are less likely than the general population to find suitable partners for marriage.	Marital preclusion
18	If I can’t control my own actions and thoughts, I am afraid that I will be laughed at.	Marital preclusion
19	If I have a family member with a mental illness, it will prevent me from finding the right person to marry.	Marital preclusion
20	If I see a psychiatrist, I’d be considered a psychopath.	Social ostracism
21	If people know I have mental problems, it would lower their opinion of me.	Marital preclusion
22	If I have a mental illness, no one would want to marry me.	Marital preclusion
23	If I have a mental illness and it is known by others, I’d be looked down upon.	Marital preclusion
24	I would be ashamed to have mental problems.	Self-deprecation
25	It’s beneath my dignity to see a psychiatrist.	Social ostracism

## Procedure

This study was approved by the Ethics Committee of the Second People’s Hospital of Guizhou Province (Approval Number: Hospital Ethics No. [2018]04). The authors were granted permission from the copyright holders to use this instrument. As this study involved human subjects, the principles of Helsinki Declaration were strictly followed. Participants were fully explained the aim and process of the study and gave oral informed consent prior to the commencement of the survey. In order to protect the privacy of the subjects, participation in this study was voluntary, anonymous and confidential, and no consent form was required. The AD8 scale was used for relatives of suspected dementia patients over 60 years old. At the end of the survey, the investigators suggested to the family members of suspected elderly dementia patients who scored 2 or more on the AD8 test, as reported by the primary caregiver, but had never visited a doctor to seek consultation from a specialist in memory disorders as soon as possible.

### Statistical analysis

Jamovi software (2.3.9; https://www.jamovi.org/download.html) and R software (R Core Team, 2022) were used for statistical analysis. Descriptive statistical methods were utilized to summarize the sociodemographic characteristics of participants, including gender, age, education level, place of residence (urban or rural), work status, and access to gaining dementia knowledge. For continuous variables, their normality was tested using the Kolmogorov–Smirnov method and was expressed as $\bar {x}\pm $ s. Non-normality data were represented by Median, *P*_25_, and *P*_75_. The Mann–Whitney U test and the Kruskal-Wallis test were employed to compare DKAS scores and PPSS scores between subgroups. If there was a statistical difference between the four groups, multiple comparisons were further performed using the Dwass-Steel-Critchlow-Fligner method. Box plots were plotted using the ggpubr package to visualize the total scores of DKAS and PPSS and the subscale scores of the participants in the four groups. In the comparison among the four groups, two-sided *p* < 0.05 was considered statistically significant; in pairwise comparisons, *p* < 0.05/6 = 0.0083 was considered statistically significant.

## Results

### Demographic characteristics of respondents

**Figure 1:**
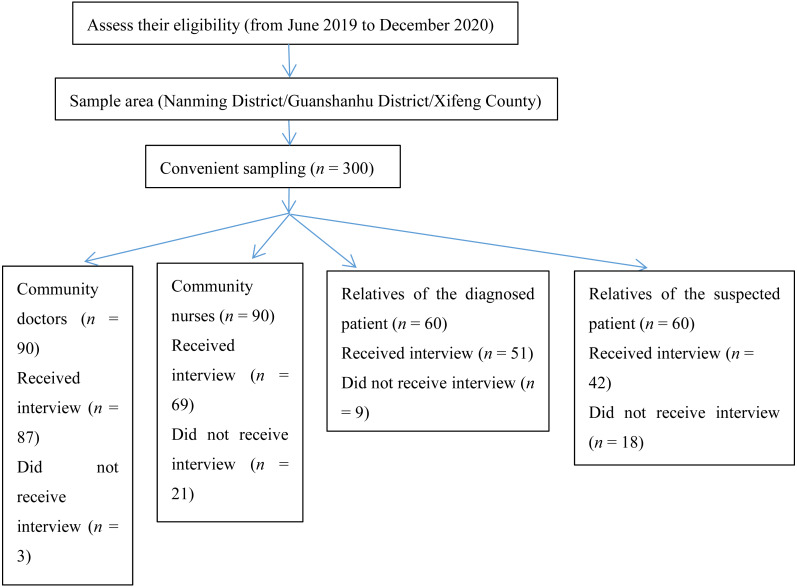
Survey flowchart of the final sample.

A total of 300 questionnaires were distributed (100 per administrative district/county), and 249 valid questionnaires were received. The effective response rate was 83.0%, with the survey dropout rate of community medical staff being 13.3% and that of relatives of diagnosed and suspected dementia patients being 22.5%. The respondents were aged between 26–70 (Median = 45 years, 39–51 years). Among them, 37.8% had secondary education or lower, and 62.2% had a junior college degree or above. 20.1% of the respondents were rural residents, and the others were urban residents. 77.9% of the respondents gained dementia knowledge through media (such as newspapers and the Internet), and 22.1% through other channels. The detailed survey flowchart is shown in Fig. 1. Table 3 shows the respondents’ demographic and professional characteristics.

**Table 3:** Demographic characteristics and their relationship to the DKAS and PPSS (*N* = 249).

Characteristics	*N* (%)	Median DKAS	*Z* or *H*	*p*	Median PPSS	*Z* or *H*	*p*
Gender			1.29	0.255		3.06	0.080
Male	109 (43.8)	38.0 (34.0–41.0)			74.0 (69.0–79.0)		
Female	140 (56.2)	38.0 (33.0–40.0)			72.0 (67.0–77.3)		
Age group			31.5	<0.001^*^		17.6	<0.001^*^
<45	123(49.4)	39.0 (37.0–41.0)			70.0 (67.0–75.0)		
≥45	126 (50.6)	36.0 (31.0–39.0)			76.0 (69.0–80.0)		
Marital status			0.08	0.778		0.99	0.319
Married	192 (77.1)	38.0 (33.8–40.0)			73.0 (68.0–78.0)		
Other states	57 (22.9)	38.0 (34.0–41.0)			73.0 (69.0–78.0)		
Work status			93.2	<0.001^*^		51.6	<0.001^*^
In-service or retired	200 (80.3)	39.0 (36.0–41.0)			71.0 (67.0–76.0)		
Unemployed or farmer	49 (19.7)	29 (27.0–32.0)			80.0 (77.0–82.0)		
Education level			107.6	<0.001^*^		50.9	<0.001^*^
Secondary education or below	94 (37.8)	32.0 (28.3–36.0)			78.0 (74.0–81.0)		
Junior college degree or above	155 (62.2)	40.0 (38.0–41.0)			70.0 (67.0–74.0)		
Group categorization			85.6	<0.001^*^		123.7	<0.001^*^
Community doctors	87 (34.9)	40.0 (38.0–42.0)			69.0 (66.0–72.0)		
Community nurses	69 (27.7)	39.0 (36.0–40.0)			69.0 (66.5–73.0)		
Relatives of the diagnosed patient	51 (20.5)	33.0 (30.0–38.0)			79.0 (76.0–82.0)		
Relatives of the suspected patient	42 (16.9)	31.5 (28.0–36.0)			80.0 (77.0–82.3)		
Place of residence			32.7	<0.001^*^		2.32	0.128
Rural	50 (20.1)	32.5 (28.0–37.0)			75.5 (68.0–80.0)		
Urban	199 (79.9)	39.0 (36.0–41.0)			72.0 (68.0–77.0)		
Per capita monthly household income			64.8	<0.001^*^		33.6	<0.001^*^
<3000 *yuan*	35 (14.1)	29.0 (26.0–31.5)			80.0 (76.5–82.0)		
≥3000 *yuan*	214 (85.9)	38.5 (36.0–41.0)			71.0 (67.3–77.0)		
Ways of gaining dementia knowledge			41.0	<0.001^*^		41.1	<0.001^*^
Media (newspapers, Internet, …)	194 (77.9)	38.5 (36.0–41.0)			71.0 (67.0–76.0)		
Other channels	55 (22.1)	32.0 (28.0–37.0)			79.0 (75.5–82.0)		

**Notes.**

Statistics was based on the Mann–Whitney *U* test (or Kruskal-Wallis test).

* *p* < 0.05

DKAS Dementia Knowledge Assessment Scale PPSS Perceived Psychiatric Stigma Scale

### DKAS scores

The median DKAS score was 38.0 (34.0–40.0). Table 4 shows that the respondents scored the highest on the “Care considerations” subscale, with a median of 12.0 (9.0–12.0); the respondents scored the lowest on the “Communication & behavior” subscale, with a median of 6.0 (4.0–6.0). A total of 44.6% of the respondents wrongly believed that dementia was a normal part of aging. About 42.2% of participants (*n* = 105) considered Alzheimer’s disease to be the most common type of dementia. Only 31.3% of the respondents thought that depressive symptoms might be mistaken for dementia symptoms. Seven demographic characteristics had high correlations with DKAS scores (Table 3). Table 4 indicates that the differences in the total DKAS scores and subscale scores of community doctors, community nurses, and relatives of diagnosed and suspected dementia patients were statistically significant (*p* < 0.05).

**Table 4:** Cohort total and subscale scores for the 25-item DKAS.

Content domain	Items	Diverse cohorts					
		Community doctors (*n* = 87) Median	Community nurses (*n* = 69) Median	Relatives of the diagnosed patient (*n* = 51) Median	Relatives of the suspected patient (*n* = 42) Median	*H*	*p*
Total DKAS score	25	40.0 (38.0–42.0)	39.0 (36.0–40.0)	33.0 (30.0–38.0)	31.5 (28.0–36.0)	85.60	<0.001^*^
Causes & characteristics	7	12.0 (10.0–14.0)	12.0 (9.0–13.0)	8.0 (6.0–12.0)	8.0 (6.0–11.0)	59.46	<0.001^*^
Communication & behavior	6	6.0 (6.0–6.0)	6.0 (4.0–6.0)	6.0 (4.0–6.0)	6.0 (3.0–6.0)	9.70	0.021^*^
Care considerations	6	12.0 (12.0–12.0)	12.0 (11.0–12.0)	10.0 (9.0–12.0)	9.0 (8.0–11.3)	59.36	<0.001^*^
Risks & health promotion	6	10.0 (10.0–11.0)	10.0 (10.0–11.0)	10.0 (7.0–10.0)	10.0 (7.0–10.0)	46.76	<0.001^*^

**Notes.**

Statistics was based on the Kruskal-Wallis test.

* *p* < 0.05

DKAS Dementia Knowledge Assessment Scale

**Figure 2:**
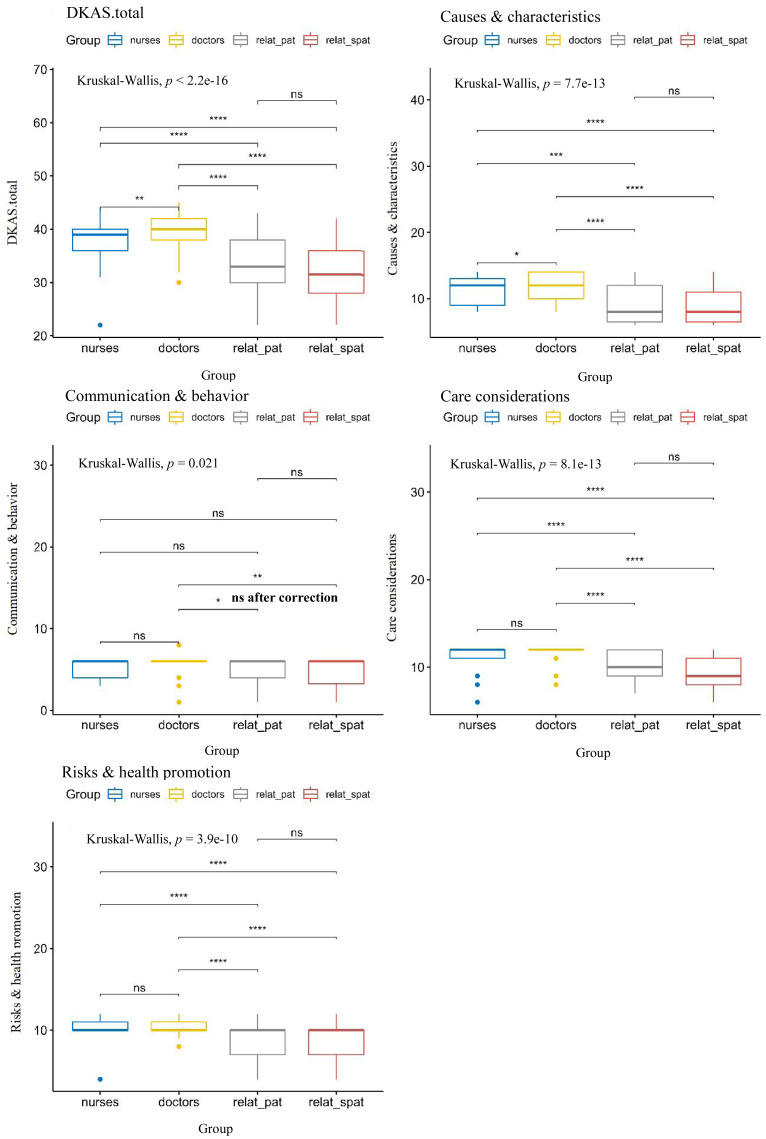
Total score and subscale scores delineated by the DKAS respondent cohort. Nurses = community nurses; doctors = community doctors; relat_pat = relatives of the diagnosed patient; relat_spat = relatives of the suspected patient. The Bonferroni correction for multiple comparisons was applied. With six group comparisons conducted, a corrected *p* of 0.0083 was required.ns: *p* > 0.05; *: *p* <  = 0.05; **: *p* <  = 0.01; ***: *p* <  = 0.001; ****: *p* <  = 0.0001. All symbols are based on the uncorrected *p*, and significant after the Bonferroni correction (*p* < 0.0083).

The Dwass-Steel-Critchlow-Fligner pairwise comparisons revealed that community doctors scored higher than community nurses, and community medical staff scored higher than the relatives of diagnosed and suspected dementia patients. The differences in the “Causes & characteristics”, “Care considerations”, and “Risks & health promotion” subscale scores between community medical staff and the relatives of diagnosed and suspected dementia patients were statistically significant ( *p* < 0.0083). The difference in the “Communication & behavior” subscale was also statistically significant between community doctors and relatives of suspected dementia patients (Fig. 2).

### PPSS scores

The median total PPSS score was 73.0(68.0–78.0). Table 5 shows that the respondents scored the highest on the “Marital preclusion” subscale, with a median of 29.0(28.0–30.0) and the lowest on the “Self-deprecation” subscale, with a median of 18.0(18.0–19.0). 27.7% of the respondents agreed that “If I have a mental illness, people will think that there is also something wrong with my ancestors”. A total of 77.1% of the respondents agreed that “If my relatives are mentally ill, I may not be able to find a suitable spouse”. There were also statistically significant differences (*p* < 0.05) in the total PPSS scores and subscale scores of community doctors, community nurses, and relatives of diagnosed and suspected dementia (Table 5).

**Table 5:** Cohort total and subscale scores for the PPSS.

Content domain	Items	Diverse cohorts					
		Community doctors (*n* = 87) Median	Community nurses (*n* = 69) Median	Relatives of the diagnosed patient (*n* = 51) Median	Relatives of the suspected patient (*n* = 42) Median	*H*	*p*
Total stigma score	25	69.0 (66.0–72.0)	69.0 (66.5–73.0)	79.0 (76.0–82.0)	80.0 (77.0–82.3)	123.7	<0.001^*^
Social ostracism	11	22.0 (20.0–24.0)	23.0 (21.0–26.0)	32.0 (29.0–34.0)	33.0 (30.0–35.0)	149.8	<0.001^*^
Marital preclusion	8	28.0 (25.0–30.0)	28.0 (25.0–30.0)	29.0 (29.0–30.0)	29.5 (29.0–30.0)	14.2	0.003^*^
Self-deprecation	6	19.0 (18.0–21.0)	18.0 (18.0–19.0)	18.0 (18.0–18.0)	18.0 (18.0–18.0)	33.1	<0.001^*^

**Notes.**

Statistics was based on the Kruskal-Wallis test.

* *p* < 0.05

PPSS Perceived Psychiatric Stigma Scale

**Figure 3:**
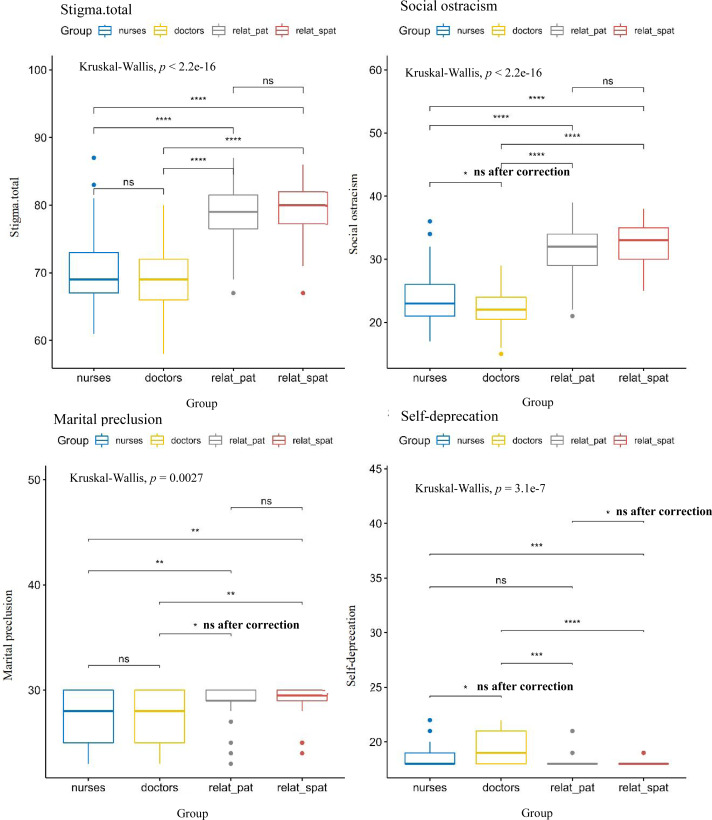
Total score and subscale scores delineated by the stigma respondent cohort. Nurses = community nurses; doctors = community doctors; relat_pat = relatives of the diagnosed patient; relat_spat = relatives of the suspected patient. The Bonferroni correction for multiple comparisons was applied. With six group comparisons conducted, a corrected *p* of 0.0083 was required.ns: *p* > 0.05; *: *p* <  = 0.05; **: *p* <  = 0.01; ***: *p* <  = 0.001; ****: *p* <  = 0.0001. All symbols are based on the uncorrected *p*, and significant after the Bonferroni correction (*p* < 0.0083).

The Dwass-Steel-Critchlow-Fligner pairwise comparisons showed that the total PPSS score and the “Social ostracism” subscale score of the relatives of diagnosed and suspected dementia patients were higher than those of community medical staff. The following differences were also statistically significant: the score differences on the “Marital preclusion” subscale between community doctors and relatives of suspected dementia patients, and between community nurses and relatives of diagnosed and suspected dementia patients; subscale scores of “Self-deprecation” between community doctors and relatives of diagnosed and suspected dementia patients, and between community nurses and relatives of suspected dementia patients (Fig. 3).

## Discussion

This is the first study in China to assess the level of dementia knowledge among three communities that are most closely related to the utilization of dementia-related health services. Our study found that the median total score of dementia knowledge among different populations in the community was 38.0(34.0–40.0). Compared with other studies, it was at a moderate level (Annear et al., 2017; Wang et al., 2018; Zhao et al., 2020). However, there were differences between different groups and subscales. Considering that 62.2% of the participants in this study had at least a junior college education, the actual total score of dementia knowledge might be lower.

In this study, the total score of dementia knowledge of participants under 45 was significantly higher than that of participants aged 45 and older. This is because younger participants have access to more sources of knowledge and are more likely to receive more updated knowledge on dementia. Our results are partially consistent with those reported by Liu et al. (2019). Higher scores were obtained by those who had junior college degrees or higher, lived in urban areas, had a high monthly income, were employed or retired, or gained dementia knowledge through media. This result can be explained by their willingness to seek professional information (such as through the newspaper, Internet, and other forms of media) and professional support (such as consulting specialist doctors and nurses). In contrast, those who had secondary education or lower, lived in rural areas, had a low monthly income, were unemployed or farmers, or gained dementia knowledge through non-media channels, had less access to dementia knowledge. Werner (2003) found that people with greater awareness of dementia symptoms were more likely to seek diagnosis as soon as possible and start continuous treatment. Therefore, demographic factors should be considered in early dementia interventions targeted at different groups.

It was found that the total DKAS scores and the scores of the “Causes & characteristics”, “Care considerations”, and “Risks & health promotion” subscales of community doctors and nurses were significantly higher than those of the relatives of diagnosed and suspected dementia patients. This is understandable because community medical staff should generally have a distinct asymmetric professional advantage. Relatives of diagnosed and suspected dementia patients in the community scored the worst on the “Communication & behavior” subscale, and the same was true for community doctors and nurses (median 6 out of 12). This result is consistent with the findings of Tierney et al. (2019), who used the DKAS to investigate 296 general practitioners. This indicates that this subscale is a key and difficult point of dementia knowledge. This subscale measures whether participants understand that “It is impossible to communicate with people with advanced dementia” and “People with advanced dementia do not respond to changes in their environment”. Community medical staff scored low on this subscale and did not show a strong professional advantage compared with the relatives of diagnosed and suspected dementia patients. Zhao et al. (2020) found in a review that the level of dementia knowledge among medical professionals in China ranged from low to medium, and this was because of limited professional training and education. Studies have shown that relatives with prior dementia training and dementia tend to have more dementia knowledge (Keogh et al., 2020). Therefore, community medical staff should pay more attention to dementia, and the understanding of dementia should be improved by promoting awareness and skill training.

According to the World Alzheimer Report 2019, more than 20% of the respondents believed that dementia is caused by external forces such as “bad luck” (21.7%), “providence” (8.7%), and “witchcraft” (1.9%) (Alzheimer’s Disease International, 2019). In a recently published systematic review of 34 studies conducted mainly in European and American countries, nearly half of the respondents believed that dementia is a normal part of aging and cannot be prevented (Garcia-Ribas et al., 2020). Our study found that 44.6% of the participants wrongly believed that dementia was part of the normal aging process, which is consistent with the findings reported in that review. Only 42.2% of our participants (*n* = 105) considered Alzheimer’s disease to be the most common type of dementia. Less than a third of the participants thought depressive symptoms could be mistaken for dementia symptoms. These findings emphasize that urgent lectures and consulting services need to be conducted to propagate large amounts of scientific dementia knowledge widely and regularly among different groups in communities in China, which has the highest number of dementia patients in the world. However, community efforts to disseminate information about dementia in China are frustrating. Publicizing the prevention and treatment of dementia in communities mainly occurs once a year during World Alzheimer’s Disease Awareness Month in September and is seldom carried out initiatively any other time.

Although Alzheimer’s disease and other forms of dementia share many symptoms (such as irritation, depression, and delusions) with psychiatric disorders like schizophrenia, they are not officially classified as psychiatric disorders (Stites et al., 2018). The psychiatric stigma associated with dementia has a significant negative impact on attitudes towards health service utilization among dementia patients, and the negative impact has extended to family caregivers of those with this label (Chang, Su & Lin, 2016). Families of dementia patients often conceal the truth, making it difficult for community medical staff to find them because “You cannot see them. They hide themselves”.

In this study, participants had a median PPSS total score of 73.0(68.0–78.0), which was moderate. This is consistent with the findings of Piver et al. (2013), who conducted a survey in France involving more than 500 people and found that the average stigma score for dementia was in the “moderate” range. We found that lower levels of education resulted in higher levels of stigma associated with dementia, which is consistent with the findings published by Lion et al. (2020). Participants who were 45 or older, unemployed or farmers, had lower monthly income, or gained dementia knowledge through non-media channels scored higher on psychiatric stigma. This may be caused by their low level of knowledge about dementia, their inability to understand dementia knowledge from the various channels, and their lack of correct understanding of public evaluations of dementia. Studies have shown that higher education and economic income contribute to lower dementia stigma (Weisman de Mamani et al., 2017), implying that future interventions should focus on dementia-related destigmatization by combining the demographic characteristics of these different populations in the community.

There was no significant difference in the total PPSS and “Social ostracism” subscale scores between the relatives of diagnosed and suspected dementia patients in the community, and between community doctors and nurses. The two groups of relatives presented higher psychiatric stigma levels than the community medical staff. This phenomenon may be significantly associated with affiliated stigma among family caregivers of dementia patients or suspected dementia patients (Van den Bossche & Schoenmakers, 2022).

Participants scored highest in the “Marital preclusion” subscale in PPSS, with relatives of diagnosed and suspected dementia patients in the community scoring higher than community medical staff. This may be related to the concern of the participants (especially relatives of diagnosed and suspected dementia patients) about the intergenerational transmission of mental illness and the “face” culture in China. Chinese have always taken family as the core since ancient times, and a person’s honor and humiliation are closely related to the family. The bad reputation of a family member impacts not only himself but also his parents, spouse, and other relatives (Yu, 2018).

We found unexpectedly that community medical staff scored higher in the “Self-deprecation” subscale in PPSS than relatives of diagnosed and suspected dementia patients, suggesting that community medical staff may have a stronger identification with negative labels such as “people with mental illness are the weak”. These intrinsic negative labels may be a significant disadvantage to tertiary dementia prevention efforts in the community.

This study has several limitations. Firstly, the cross-sectional design of this study revealed only the associations rather than causality among variables. Secondly, due to the limited conditions, this study did not include community groups without diagnosed or suspected dementia patients in the family (this group may not share the same concerns about dementia). The findings for community populations in the three selected districts and counties may not reflect the situation in Guizhou Province or other regions in China, so the research results cannot be extended to all regions in China. Thirdly, the sample size of the four groups of people in this study was small. Due to the impact of COVID-19, entry into communities was strictly controlled, which to some extent affected, the expected number of participants. Fourthly, because there is no gold standard for assessing dementia stigma (Noguchi et al., 2022), in our study, participants filled out a scale for measuring psychiatric stigma in healthy people to understand the level of dementia-related psychiatric stigma in different groups of the community. The assessment of dementia-related psychiatric stigma was based on self-reported questionnaires, which may not reflect the true severity of dementia-related stigma. Future studies need to further expand the sample size of rural communities and investigate the broader general population.

## Conclusions

There is a significant imbalance in the level of dementia knowledge and psychiatric stigma within different community populations. We found that community doctors and nurses did not show a complete professional advantage in dementia knowledge compared with relatives of diagnosed and suspected dementia patients, and they even showed higher psychiatric stigma in some subscales. To improve the level of dementia knowledge among different populations in the community and reduce the stigma associated with dementia, we suggest the following methods: (1) Raise public awareness of dementia through the media and spread dementia-related information in a more responsible manner. (2) Formally include the prevention and treatment of dementia in China’s basic public health services. (3) Secondary and tertiary medical institutions should undertake long-term special training on dementia prevention and treatment techniques with initiative; primary medical institutions should improve the accessibility of community services (Quail et al., 2020). (4) Conduct educational programs to prevent and destigmatize dementia targeted at people aged 45 and older and those in communities with low income and low education. (5) Current evidence suggests that negative and stigmatizing attitudes towards dementia may develop at a young age (Farina et al., 2020); therefore, the knowledge of dementia prevention and control should be included in middle school curriculums in China to improve the understanding of dementia.

##  Supplemental Information

10.7717/peerj.14613/supp-1 Data S1 Raw data

## Additional Information and Declarations

### Data Availability

The following information was supplied regarding data availability: The raw data is available in the Supplemental Files.
