# Supplementary material for: Senile dementia and psychiatric stigma among community health service providers and relatives of diagnosed and suspected dementia patients: a cross-sectional study
Source: PeerJ. 2023 Jan 11;11:e14613. doi: 10.7717/peerj.14613 (PMC9840390; doi:10.7717/peerj.14613)
Supplement: Data S1 [file peerj-11-14613-s001.zip › raw data or code files 2..docx]

**List of raw data variable assignments**

| Variable | assignment |
| --- | --- |
| sex | Male =1  Female=2 |
| Age (years) | actual value |
| Age group | ＜45=1  ≧45=2 |
| Marital status | Married=1  Other states=2 |
| Group categorization | Community doctors=1  Community nurses=2  relatives of patient=3  Suspected dementia relatives=4 |
| Work status | In-service or retired=1  Unemployed or farming=2 |
| Education level | Secondary education and or below=1  Junior college degree or above=2 |
| Place of residence | Rural=1  Urban=2 |
| Per capita monlthy household income | ＜3000 yuan=1  ≧3000 yuan=2 |
| Ways understand dementia | Media (newspapers, Internet, etc.)=1  Other Way=2 |
| Total DKAS score | actual value |
| Causes & characteristics | actual value |
| Communication & behaviors | actual value |
| Care considerations | actual value |
| Risks & health promotion | actual value |
| Total stigma score | actual value |
| Social ostracism | actual value |
| Marital preclusion | actual value |
| Self-deprecation | actual value |
